# Inclusion of abortion-related care in national health benefit packages: results from a WHO global survey

**DOI:** 10.1136/bmjgh-2023-012321

**Published:** 2023-08-29

**Authors:** Katy Footman, Kratu Goel, Ulrika Rehnström Loi, Andrew J Mirelman, Veloshnee Govender, Bela Ganatra

**Affiliations:** 1 UNDP–UNFPA–UNICEF–WHO–World Bank Special Programme of Research, Development and Research Training in Human Reproduction (HRP), Department of Sexual and Reproductive Health and Research, WHO, Geneva, Switzerland; 2 Department of Health Financing and Economics, WHO, Geneva, Switzerland

**Keywords:** health insurance, health systems, maternal health, cross-sectional survey

## Abstract

**Introduction:**

Service inclusion in a country’s health benefit package (HBP) is an important milestone towards universal health coverage. This study aimed to explore HBP inclusion of abortion interventions globally.

**Methods:**

Secondary analysis of the WHO HBP survey, in which officially nominated survey focal points were asked which interventions were included within the HBP of their country or area’s largest government health financing scheme. Abortion inclusion was compared by region, income, legal status of abortion and HBP design process variables. Abortion inclusion was compared with other sexual and reproductive health (SRH) services.

**Results:**

Below half (45%) reported that abortion is included, but treatment of complications from unsafe abortion was more commonly included (63%). Fewer fully included essential abortion medications (22% mifepristone, 42% misoprostol). Abortion was less commonly included than any other SRH service in the survey. Unlike most SRH services, higher cost, higher technology care to treat complications of unsafe abortion was more commonly included than the relatively lower cost, lower technology service of induced abortion. Higher-income contexts and less restrictive legal environments had higher abortion inclusion. Some contexts had additional restrictions, with abortion inclusion dependent on the patient’s reason for seeking care.

**Conclusion:**

This global survey finds that abortion services and medications are often not included within HBPs, while treatment of complications from unsafe abortion is more commonly included. There are opportunities to improve HBP abortion inclusion across different legal contexts, which can improve health outcomes and reduce the need for higher cost treatment of complications from unsafe abortion.

**WHAT IS ALREADY KNOWN ON THIS TOPIC**

- Abortion is often excluded from health benefit packages (HBPs), but there is a lack of comprehensive data on the inclusion of abortion within HBPs.

**WHAT THIS STUDY ADDS**

- This study provides the most thorough assessment to date of the inclusion of comprehensive abortion care and abortion medications within HBPs. The survey also allowed comparison of abortion with other sexual and reproductive health services, and assessment of the association between HBP abortion inclusion and characteristics of the HBP design process.

**HOW THIS STUDY MIGHT AFFECT RESEARCH, PRACTICE OR POLICY**

- There is a clear need for abortion to be prioritised for inclusion within discussions about universal health coverage and within HBPs, across a range of legal contexts for abortion.

## Background

Of the estimated 73 million abortions that occur annually,^1^ almost half (45%) are unsafe,^2^ creating a high burden of complications, maternal deaths and costs for individuals, families and health systems.^2^ Legal restrictions on safe abortion are the leading cause of unsafe abortion, but people living in countries with permissive abortion laws may still lack access to affordable abortion services and medications.^3 4^ Despite the relatively low health system costs of safe abortion services in most contexts,^5^ even low patient costs can prevent or delay care-seeking for populations that are socially or economically disadvantaged,^6–8^ particularly when limited service availability means patients face additional indirect costs of travel.^9^ These patient costs may increase due to stigma,^10^ as the need for secrecy may reduce access to financial resources from family or friends,^11^ may increase the need to travel or may fuel requests for unofficial payments by healthcare providers.^12^ The health system and patient costs of treating complications from unsafe abortion are higher on average than the costs of providing a safe abortion,^5^ and out-of-pocket expenditure on postabortion care for complications can cause catastrophic expenditure(s), where individuals and families are pushed further into debt and poverty in order to pay for care.^9 13^

Universal health coverage (UHC) means that all individuals receive the health services they need without facing financial hardship.^14^ Increasingly, it is acknowledged that progress towards universal sexual and reproductive health and rights (SRHR) is not possible without progress towards UHC,^8^ but also that SRHR is a fundamental aspect of UHC.^15–17^ Although a strong case has been made for prioritising SRHR interventions and services in UHC, politics often play a significant role in priority-setting for health.^5^ This can put services for historically marginalised populations (eg, youth) or stigmatised services (eg, safe abortion) at risk.^5^ Cultural and political sensitivities related to sexuality, reproductive choice and gender equality can all constrain the inclusion of comprehensive SRHR in UHC while also creating policy, regulatory and legislative barriers to providing and accessing such services.^17^ Some sexual and reproductive health (SRH) services have therefore not always been included within UHC-related decisions about health financing, resource allocation and priority-setting,^8^ and abortion is often excluded from monitoring indicators for UHC.^18^

A health benefit package (HBP), defined as ‘a set of services that can be feasibly financed and provided under the actual circumstances in which a given country finds itself’,^19^ is essential for creating a sustainable UHC system in the context of limited resources. The HBP defines what services can and will be offered, and to whom,^20^ and a range of mechanisms exist for setting these priorities. There are few studies on the inclusion of SRH services in health financing schemes.^8^ Previous assessments of abortion’s inclusion within HBPs have been limited by their reliance on publicly available information,^10^ exclusion of countries with restrictive legal environments^4^ or inclusion of only one subregion.^21^ Some studies have also assessed inclusion of abortion within the essential package of health services (EPHS),^15 22^ which acts as a policy statement of intent and commitment but does not tell us whether an intervention is feasibly financed.^19^

In 2020/2021, the WHO included questions about inclusion of abortion and other SRH services within a global survey on HBP. These survey data provide information on the inclusion of abortion interventions within the HBP of the largest government health financing scheme in each country or area that responded to the survey from WHO. The survey provides the most comprehensive assessment of the inclusion of abortion within HBPs to date. (The term ‘countries’ or ‘national’ should be understood to refer to countries and areas. The designations employed and the presentation of the material in this platform do not imply the expression of any opinion whatsoever on the part of WHO concerning the legal status of any country, territory, city or area or of its authorities, or concerning the delimitation of its frontiers or boundaries.)

The specific objectives of this paper are (1) to examine the reported inclusion of abortion and treatment of abortion complications within the HBP of the largest government-financed scheme in each country or area, including additional details from free-text responses; (2) to assess the reported inclusion of abortion medications in these HBPs; (3) to compare different patterns of HBP inclusion between geographical regions, country income statuses, and policy and regulatory contexts for abortion; (4) to understand potential links between HBP design processes and the inclusion of abortion within HBPs; and (5) to compare the inclusion of abortion with other SRH services and assess the potential reasons for exclusion.

## Methods

### HBP survey

The methodology of the 2020/2021 HBP survey^23^ is described in detail elsewhere. In brief, the survey was sent by email in October 2020 to officially nominated survey focal points in member states using a WHO online platform. Respondents were prompted to collaborate with other officials and agencies that have expert knowledge if needed. Regular reminders were sent to respondents and responses were accepted until mid-2021. The survey was estimated to take 60–90 min to complete. The survey was available in English, French, Spanish, Arabic and Russian.

At the start of the HBP module, respondents were asked to identify and rank the five largest government health financing schemes in the country or area, with ‘largest’ specified as referring to the size of population eligible to receive services. Government health financing schemes were specified as any public sector scheme for health insurance or service provision which includes coverage for groups such as the general population, public sector employees and the military. Respondents were asked to select schemes that included a range of services, if possible, rather than vertical schemes that focused on one disease or intervention. Respondents were asked to provide details about the largest scheme, such as whether it was national or subnational, the population coverage, type of coverage, the process for determining service inclusion and conditions of coverage.

Participants were then asked which general interventions, condition-specific interventions and medications were included within the HBP of the largest of these government schemes. For condition-specific interventions, respondents were asked to indicate which interventions were included in the largest HBP across 37 programme areas. There were five programme areas listed under an ‘SRHR’ section, which included contraception, sexual health, abortion care, female genital mutilation and gender-based violence. Other relevant SRHR programme areas included in the survey were maternal health, cervical cancer and HIV. As we were not able to ask about every possible intervention for each programme area, we selected two to five interventions for each area, which acted as ‘tracers’, to give a proxy indication of the extent of intervention coverage. Four tracer interventions were included for most programme areas, though there were five for contraception and two for gender-based violence. All tracer interventions were ordered so that they ranged from lowest cost, lowest technology (tracer 1) to highest cost, highest technology (tracer 4), though costs and technology levels were not intended to be comparable across programme areas. For abortion care, the four tracer interventions were (1) induced abortion under 12 weeks, (2) induced abortion over 12 weeks, (3) care for miscarriage or incomplete abortion, and (4) care for complications of unsafe abortion.

### Measures

HBP inclusion was assessed for each of the four abortion tracer interventions, for a derived summary indicator of ‘any induced abortion’ (inclusion of tracer 1 and/or tracer 2) and a derived summary indicator of ‘any abortion-related care’ (inclusion of any of the tracers 1–4).

To assess how HBP inclusion of abortion services and medications varies between countries with different legal and policy contexts for abortion, we used Global Abortion Policies Database (GAPD) data (https://abortion-policies.srhr.org/, downloaded on 24 August 2022). GAPD data on the legal grounds for abortion were used to categorise countries into five groupings based on the legal indications under which a person can have an abortion in each country: on request; on specified grounds I (social or economic reasons, foetal impairment, rape, incest, intellectual or cognitive disability of the woman, and mental health); on specified grounds II (physical health, health or life only); no specified grounds; or varies by jurisdiction. If the abortion law varies by jurisdiction but each jurisdiction’s law fell into the same category, then that category was used instead (eg, Nigeria and UK). GAPD data were also used to compare HBP inclusion of medication abortion against the inclusion of these medications in the country’s Essential Medicines List (EML).

The World Bank country income categories were used to group countries by income status,^24^ and the Sustainable Development Goal regional categorisation was used.^25^

### Analysis

Data were analysed descriptively in Stata V.17 to assess HBP inclusion of abortion by region, income status and legal status of abortion. Scheme characteristics were assessed for countries that include any induced abortion. HBP inclusion of abortion was compared with inclusion of other tracer SRH interventions. HBP inclusion of abortion medications was compared with inclusion of other SRH medications. Abortion medication inclusion was also assessed by its status on the country’s EML and by HBP inclusion of any induced abortion.

The association between characteristics of the HBP design process and HBP inclusion of abortion was also assessed. These HBP design process variables included whether decision making around the HBP is linked to a formalised health technology assessment (HTA) process (HTA processes have existed for several decades and are mechanisms to implement evidence-based decision making in the health sector. HTA is defined as ‘a multidisciplinary process that uses explicit methods to determine the value of a health technology at different points in its lifecycle. The purpose is to inform decision-making in order to promote an equitable, efficient, and high-quality health system’.),^26^ whether decisions are conducted in collaboration with organisations external to the country, whether pre-existing lists or frames of services are used to inform HBP inclusion of SRH interventions, and whether periodic revisions are made to the contents of the HBP. In a first step, each variable for the HBP design process was cross-tabulated with HBP inclusion of any induced abortion, and the association was assessed using χ^2^ tests. A multivariate logistic regression model with all design process variables was then run (excluding the pre-existing lists or frames variable due to high levels of missing data (n=49)). Potential explanatory variables (region, income and abortion legal status) were added sequentially to assess whether any significant association between design process and HBP abortion inclusion remained.

### Patient and public involvement

Patients and the public were not involved in the design, conduct, reporting or dissemination plans of this analysis.

## Results

### Inclusion of abortion within HBPs

The HBP survey was completed by 115 countries and areas, giving a global response rate of 58%. However, only 112 completed the section on condition-specific interventions, and 114 completed the section on medications. Countries or areas that completed the HBP survey are estimated to include 80% of the global population.

**Figure 1:**
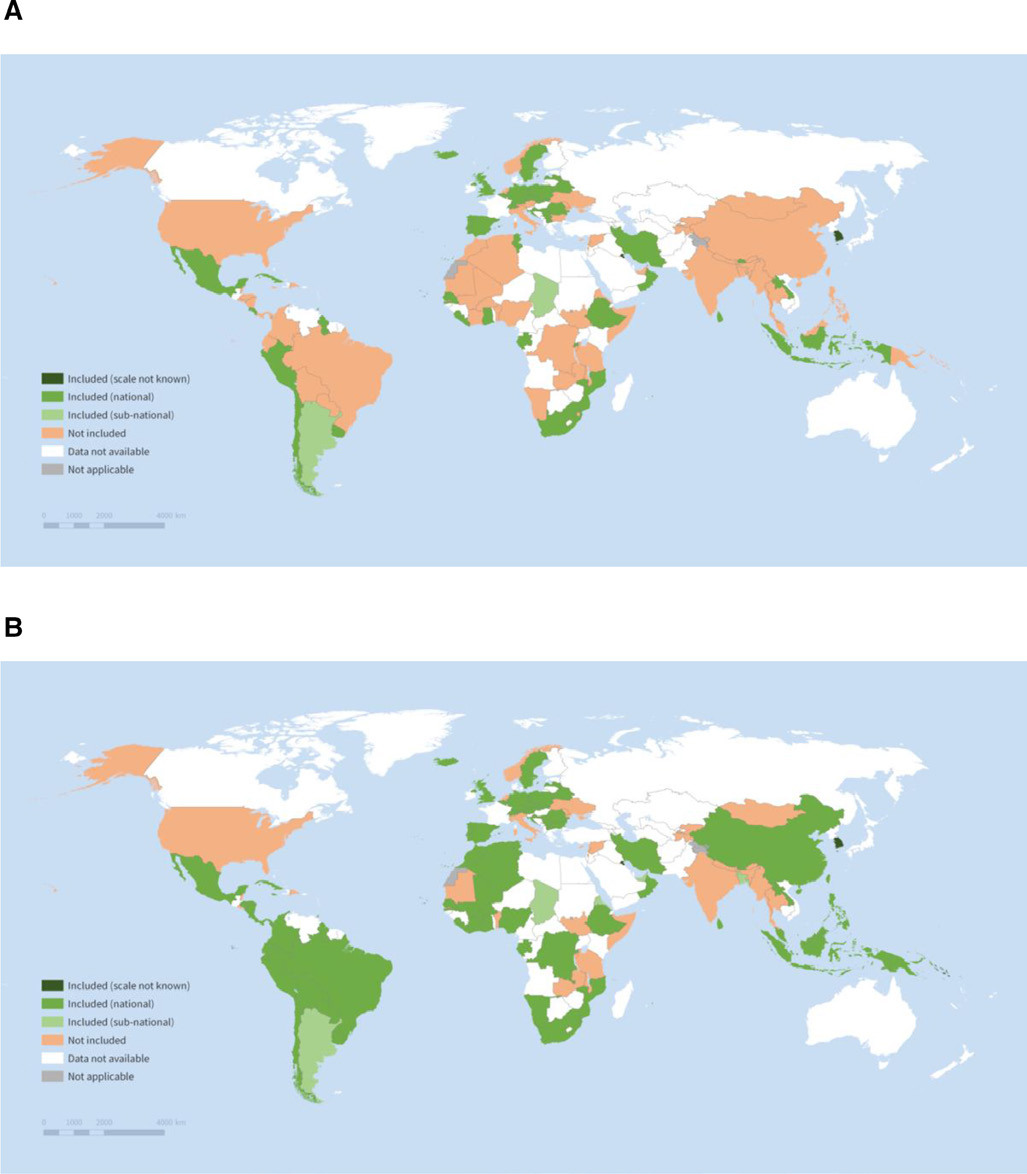
Inclusion of (A) any induced abortion care and (B) any abortion-related care in the HBP of the largest public-financed scheme. The designations employed and the presentation of the material in this publication do not imply the expression of any opinion whatsoever on the part of WHO concerning the legal status of any country, territory city or area or of its authorities, or concerning the delimitation of its frontiers or boundaries. Dotted and dashed lines on maps represent approximate borderlines for which there may not yet be full agreement. Source: WHO 2020/21 Health Benefit Package Survey.

In total, 50 countries out of 112 with data available on condition-specific interventions (45%) reported that induced abortion under 12 weeks was included in the HBP of their largest government-financed scheme (table 1). Fewer (n=41, 37%) countries reported abortion over 12 weeks was included. Care for miscarriage or incomplete abortion (n=74, 66%) or for complications of unsafe abortion (n=71, 63%) was more commonly included. HBP inclusion of any induced abortion and any abortion-related care by country is shown in figure 1.

**Table 1:** Inclusion of abortion-related services in the HBP of the largest public-financed scheme, by region, income and legal context

	Areas and countries	Induced abortion under 12 weeks		Induced abortion over 12 weeks		Care for miscarriage or incomplete abortion		Care for complications of unsafe abortion	
	N	n	%	n	%	n	%	n	%
All countries and areas	112	50	45	41	37	74	66	71	63
Region									
Sub-Saharan Africa	30	13	43	9	30	19	63	16	53
Northern Africa and Western Asia	10	3	30	2	20	5	50	6	60
Central and Southern Asia	9	4	44	4	44	4	44	5	56
Eastern and South-Eastern Asia	11	4	36	3	27	6	55	5	45
Europe and Northern America	27	17	63	14	52	19	70	18	67
Latin America and the Caribbean	21	9	43	9	43	18	86	18	86
Oceania	4	0	0	0	0	3	75	3	75
Income status									
Low income	17	6	35	4	24	9	53	8	47
Lower middle income	37	11	30	8	22	21	57	21	57
Upper middle income	27	13	48	13	48	22	81	21	78
High income	31	20	65	16	52	22	71	21	68
Abortion legal status									
On request	32	21	66	16	50	22	69	21	66
Grounds specified I	45	21	47	18	40	34	76	33	73
Grounds specified II	23	5	22	4	17	9	39	10	43
No grounds specified	9	2	22	2	22	7	78	5	56
Varies by jurisdiction	3	1	33	1	33	2	67	2	67

Grounds specified I: legal grounds for abortion include indications of social or economic reasons, foetal impairment, rape, incest, intellectual or cognitive disability of the woman or mental health. Grounds specified II: only legal grounds for abortion are life, physical health or health. Survey reports of inclusion within the HBP imply an ex-ante promise to provide a particular service or intervention as part of the benefits package,but not the actual provision of that service itself.

HBP, health benefit package.

HBP inclusion of induced abortion was highest in Europe and Northern America and lowest in Oceania, though only four countries in this region completed the survey (table 1). Care for complications of unsafe abortion or treatment of miscarriage and incomplete abortion were more commonly included than induced abortion in almost every region, except for Central and Southern Asia, and Europe and North America. By income status, there was greater HBP inclusion of each abortion intervention in higher-income countries than lower-income countries.

HBP inclusion of induced abortion care was lowest in countries with no legal grounds for abortion or where the only legal grounds were life, health or physical health (grounds specified II). HBP inclusion was higher in countries with other legal grounds for abortion such as mental health, social or economic reasons, rape, incest, foetal indications or disability (grounds specified I), and inclusion was highest where abortion is legal on request. However, two countries with no legal grounds specified for abortion (Senegal and Sierra Leone) reported that abortion is included (online supplemental appendix 1). Conversely, 11 countries where abortion is legal on request reported that abortion is not included (Bulgaria, China, Kyrgyzstan, Mongolia, Nepal, Norway, Republic of Moldova, San Marino, Singapore, Thailand and Ukraine). Countries where the abortion law varies by jurisdiction also had low HBP inclusion of any induced abortion care. For care for miscarriage or incomplete abortion or for complications from unsafe abortion, there was no clear relationship between HBP inclusion and the legal status of abortion, but HBP inclusion was lowest where the only legal grounds for abortion were life, health or physical health (grounds specified II).

10.1136/bmjgh-2023-012321.supp1 Supplementary data

### Inclusion of abortion medications within HBPs

HBP inclusion of SRH medications within the largest government-financed scheme was also assessed (n=114). Medications which are only indicated for abortion (mifepristone (22%) and the mifepristone-misoprostol combined regimen (22%)) were the least commonly fully included SRH medications (online supplemental appendix 2). Misoprostol, which is used for abortion and postabortion care, but also for treatment of postpartum haemorrhage and non-SRH indications (gastric ulcer), was more commonly fully included (42%), though less commonly than other SRH medications such as oxytocin (66%), magnesium sulfate (62%) and folic acid (63%) (online supplemental appendix 2).

Full or partial inclusion of misoprostol within the HBP was higher in countries where misoprostol is included in the EML, and HBP inclusion of mifepristone or the combi-pack was also higher where the combi-pack is included in the EML (table 2). Five countries that included misoprostol in their EML did not include the medication in their HBP: Bangladesh, Latvia, Norway, Poland and Slovakia. There were also five countries that included the combi-pack in their EML but did not include mifepristone or the combi-pack in their HBP: Colombia, Latvia, North Macedonia, Norway and Sweden.

**Table 2:** HBP inclusion of abortion medications in the largest public-financed scheme by EML status and by HBP inclusion of any induced abortion care (N=114)

HBP inclusion of misoprostol by misoprostol EML status (n=68*)				
	**EML does not include misoprostol† (n=19**)		**EML does include misoprostol† (n=49**)	
	**n**	**%**	**n**	**%**
Fully or partially included	11	58	44	90
Not included	8	42	5	10
**HBP inclusion of mifepristone or combi-pack, by combi-pack EML status (n=52)‡**				
	**EML does not include combi-pack (n=28**)		**EML does include combi-pack (n=24**)	
	**n**	**%**	**n**	**%**
Fully or partially included	16	57	19	79
Not included	12	43	5	21
**HBP inclusion of SRH medications in countries and areas where any induced abortion care is included in the largest public-financed scheme’s HBP (n=50)§**				
	**Misoprostol (n=41**)		**Mifepristone or combi-pack (n=33**)	
	**n**	**%**	**n**	**%**
Fully or partially included	35	85	25	76
Not included	6	15	8	24

*High levels of missing data for EML status (n=15) and misoprostol inclusion (n=34).

†For gynaecological indications or indications are not specified.

‡High levels of missing data for EML status (n=15) and mifepristone or combi-pack inclusion (n=51).

§Missing data for misoprostol inclusion (n=9) and mifepristone or combi-pack inclusion (n=17).

EML, Essential Medicines List; HBP, health benefit package; SRH, sexual and reproductive health.

Six countries where induced abortion was included in the largest government-financed scheme did not fully or partially include misoprostol, mifepristone or the combi-pack (table 2): Costa Rica, Latvia, North Macedonia, Poland, Serbia and Slovakia. Peru and the Republic of Korea did include misoprostol but did not include mifepristone or the combi-pack.

### Characteristics of government-financed schemes that included abortion

Among countries that included induced abortion within the HBP of their largest government-financed scheme (n=50), 45 schemes were national, while two were subnational and the remainder were missing (n=3) (figure 1). Almost all (n=48) covered more than 50% of the population (data not shown). Among schemes with HBPs that included any induced abortion, the most common type of coverage was automatic based on residence (n=27), followed by mandatory, payment-based coverage (n=22), and the least common was voluntary payment-based coverage (n=11) (coverage types were not mutually exclusive).

Most schemes had conditions restricting access to coverage of any health interventions within the HBP. For schemes that included abortion, these conditions included which population groups can access services (n=39), which interventions can be delivered (eg, physician gatekeeping) (n=33), cost-sharing arrangements (n=30), which interventions are available (eg, low-cost, generic) (n=27) and waiting times to receive services (n=25). However, these conditions were not abortion-specific.

Free-text responses to the questions about HBP inclusion of abortion were provided by 27 countries and areas (online supplemental appendix 3). In nine cases, these responses noted additional conditions for abortion inclusion. The free-text response for Latvia noted that abortion is paid for up to 12 weeks if the pregnancy is a result of rape, while state-paid abortion is performed up to 24 weeks for medical indications. The free-text response for Poland explained that abortion is included, regardless of gestation, if the woman’s life or health is endangered by the pregnancy or when the pregnancy is the result of a criminal act. For Peru, the response noted that abortions are included up to 22 weeks for therapeutic reasons when it is the only way to save the life of the pregnant woman. Other responses specified that abortion is included in the HBP if it is for medical indications (Maldives, occupied Palestinian territory, including east Jerusalem), including contexts where abortion is legal on request (North Macedonia, Slovakia). Ukraine’s response specified that abortions were only for medical and social reasons, while Tunisia’s response highlighted that abortion was only included in the HBP within public health (Ministry of Health) structures.

Importantly, two free-text responses also highlighted that while abortion is not specifically included in the HBP of government health financing schemes, abortion is covered by the public sector through a sexual and reproductive health programme or national family planning service (Nepal, Chile). In other cases (n=11), free-text responses were used to provide a link to an information page about abortion in the country and to clarify the legal status of abortion, or in three cases to explain that no documented information was found (Plurinational State of Bolivia, El Salvador and Dominican Republic).

### Implications of HBP design processes for inclusion of abortion in HBPs

Table 3 compares HBP inclusion of abortion by HBP design process variables. HBP inclusion of any induced abortion and of any abortion-related care was higher in countries where decisions around the HBP are linked to a formalised HTA process, where HBP decisions are not conducted in collaboration with external organisations, where pre-existing lists or frames of services are used to inform HBP inclusion of SRH interventions, and where periodic revisions are made to the content of the HBP. However, only the association between HBP inclusion of abortion and HBP decisions being linked to a formalised HTA process was statistically significant. After adjusting for region, country income status and legal status of abortion using multivariable logistic regression, the association between HBP inclusion of abortion and whether HBP decisions were linked to a formalised HTA process was no longer significant (data not shown).

**Table 3:** Proportion of HBPs that include abortion-related services in the largest government-financed scheme, by HBP design process variables (N=112)

	Proportion with service included in HBP	
	Include any induced abortion (%)	Include any abortion-related care (%)
Are decisions around the benefit package linked to a formalised HTA process?§		
Yes	**61**	79
No	**37**	67
Are benefit package decisions or processes conducted in collaboration with organisations external to your country?*		
Yes	40	64
No	49	77
Do you use any pre-existing lists or frames of services to inform the coverage of sexual and reproductive health interventions in your benefit package? (eg, the Minimum Initial Service Package (MISP) for sexual and reproductive health)†		
Yes	55	82
No	40	60
Are there periodic revisions to the contents of the HBP?‡		
Yes	48	71
No	39	75

Numbers in bold indicate p<0.05 using a χ^2^ test.

*11 responses missing.

†49 responses missing.

‡13 responses missing.

§17 responses missing.

HBP, health benefit package; HTA, health technology assessment.

### Comparison to other SRH and newborn health tracer interventions

**Figure 2:**
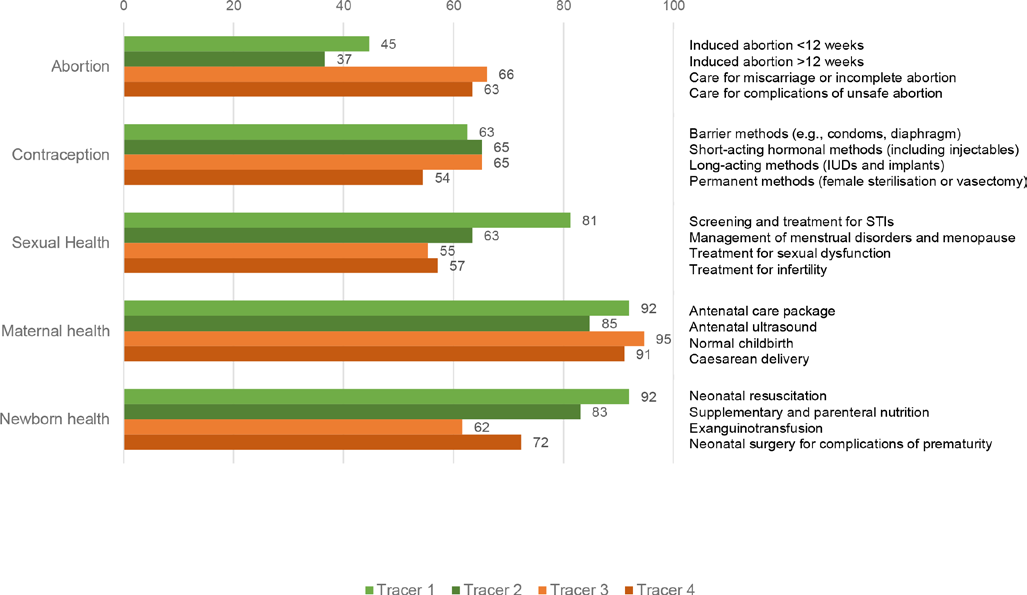
Proportion of countries or areas that include sexual and reproductive health tracer interventions in the health benefit package of the largest government-financed scheme from tracer 1 (lowest cost, lowest technology) to tracer 4 (highest cost, highest technology). IUD, intrauterine device; STI, sexually transmitted infection.

In figure 2, inclusion of the four abortion tracer interventions within the HBP of the largest government-financed scheme is compared with inclusion of the tracer interventions for other SRH and newborn health services. The first tracer item for abortion (induced abortion<12 weeks) was included in substantially fewer HBPs (45%) than the first tracer item in any other SRH area (63%–92%). For abortion care, the lower cost, lower technology tracer items (induced abortion under and over 12 weeks) were less commonly included than the higher cost, higher technology tracer items (treatment for miscarriage or incomplete abortion and treatment of complications of unsafe abortion). In sexual and newborn health, the reverse pattern was seen, with lower cost, lower technology interventions more commonly included. For contraception, long-acting method inclusion was similar to lower cost short-acting or barrier contraceptive methods. For maternal health, normal childbirth and caesarean delivery were included in almost all countries, as were the lower cost, lower technology antenatal services.

### Reasons for exclusion of SRH services

Respondents were asked about the reasons for exclusion of any specific SRH services from the HBP (table 4). The most common reason respondents selected was that there was insufficient evidence to meet the requirements of the priority-setting criteria for the HBP (18%). This was followed by services being politically contested (12%), donor funding (10%), and insufficient participation or exclusion of relevant stakeholders (8%), but most did not respond. This question referred to SRH services broadly rather than abortion specifically, but in the free-text responses, legal restrictions on abortion were commonly mentioned. Other free-text responses mentioned religious and cultural reasons, that SRH services are not relevant in their setting, and that the HBP only includes services aimed at prevention and treatment of disease. Reasons for exclusion of SRH services from the HBP did not vary significantly between countries where induced abortion is included versus not included. However, political reasons were slightly more commonly cited (13% vs 10%) and donor involvement was slightly less commonly cited (8% vs 12%) in countries where abortion is not included. Only nine countries had no legal grounds for abortion, but a higher proportion (33%) of responses from these contexts stated that SRH services were excluded because they were politically contested.

**Table 4:** Reasons for exclusion of specific SRH services from the largest government-financed scheme’s HBP (N=112)

	Politically contested	Insufficient evidence to meet the requirements of the priority-setting criteria	Insufficient participation or exclusion of relevant stakeholders	Excluded because it was funded by donors
Overall % answering ‘Yes’	12	18	8	10
% answering yes by HBP inclusion of abortion				
Countries where induced abortion is included	10	18	8	12
Countries where induced abortion is not included	13	18	8	8
% answering yes by abortion legal status				
On request	13	22	3	9
Grounds specified I (any other indication)	9	16	7	11
Grounds specified II (life, health, physical health only)	9	17	13	4
No grounds specified	33	11	11	11
Varies by jurisdiction	0	33	33	33
Non-response (n)	15	13	17	17

Grounds specified I: legal grounds for abortion include indications of social or economic reasons, foetal impairment, rape, incest, intellectual or cognitive disability of the woman or mental health. Grounds specified II: only legal grounds for abortion are life, physical health or health. In the survey, respondents were asked to indicate what the reasons were for exclusion of specific SRH services from the HBP, and each column in this table was a response option. The response option ‘politically contested’ was intended to indicate whether SRH services were excluded because they are politically contested. The response option ‘insufficient evidence to meet the requirements of the priority-setting criteria’ was intended to indicate whether SRH services were excluded because there is insufficient evidence (eg, of health impact or cost-effectiveness) to justify including the service in the HBP. ‘Insufficient participation or exclusion of relevant stakeholders’ was intended to indicate whether services were excluded because stakeholders with relevant knowledge or interest in SRH may have been excluded from the process. The response option ‘excluded because funded by donors’ was intended to indicate whether SRH services were excluded for this reason.

HBP, health benefit package; SRH, sexual and reproductive health.

## Discussion

This study provides a comprehensive assessment of the inclusion of induced abortion, postabortion care and abortion medications within the HBPs of the largest government health financing schemes in 114 countries and areas. The findings highlight that comprehensive abortion care and abortion medications are not included within HBPs in most countries, which has important implications for the accessibility and affordability of abortion. Future inclusion of abortion care within HBPs could markedly reduce health system expenditure on emergency postabortion care for complications from unsafe abortion: the health system cost of postabortion care in low-income and middle-income countries was estimated at $232 million in 2014, which could be reduced to $20 million if all abortions occurred under safe conditions.^27^ Recent estimates indicate that the direct health system cost of providing a safe abortion service in low-income and middle-income countries is US$12, while the average direct cost of providing postabortion care for shock, sepsis, uterine perforation or haemorrhage is US$75.^5^ Beyond the economic rationale, improving HBP inclusion of abortion care can more importantly help remove barriers to affordable abortion care and reduce the social and health impacts of unsafe abortion.^10^

The findings from this analysis support and extend those of previous research, which has similarly identified low inclusion of abortion in HBPs, including in those countries with permissive abortion laws.^4^ The GAPD, which uses document review methods to extract data from explicit text of laws, policies or guidelines, identified 17 countries (out of 41 with information available) that include abortion for all individuals regardless of indication.^10^ Another study used online resources and an email-based survey among reproductive health experts to determine public funding policies for abortion in countries where abortion is permitted for economic or social reasons, or on request.^4^ This analysis identified that 34 out of 80 countries with liberal abortion laws had full funding for abortion, while 25 had partial and 21 had no funding.^4^ A review of six Asia Pacific countries found only one (Thailand) included safe abortion in the HBP, despite other countries in the region having relatively liberal abortion laws.^21^ Past studies have identified that abortion is also rarely included within EPHS policy statements.^15 22^

In this study, inclusion of abortion within HBPs was higher in countries with less legally restrictive environments for abortion. However, findings suggest that even where abortion can be legally provided for certain indications, or where abortion medications are included in the EML, the lack of inclusion with HBPs may impede access. Unlike most other SRH services, lower cost, lower technology abortion services are less likely to be included within HBPs than higher cost postabortion care, and political considerations were cited as a reason for exclusion of SRH services from HBPs in several countries. This echoes previous research which illustrated how abortion stigma can influence the inclusion of abortion within UHC priorities.^28^ However, there has been recent progress towards including abortion within health financing mechanisms in countries with diverse legal contexts for abortion and varied sociocultural and religious environments, highlighting opportunities to expand affordable access to safe abortion care.^29^ Lessons learnt from these experiences include the need for close partnership between national and regional health structures and civil society, local evidence on costing, and an emphasis on safe technologies, task-shifting and referral linkages.^29^ These lessons mirror the key policy actions identified by WHO for ensuring that SRH services are comprehensively integrated within HBPs: transparent and inclusive engagement of stakeholders, evidence-based priority-setting, inclusive priority-setting criteria, and the use of evidence and strategic reframing to advocate for prioritisation of contested SRH services.^19^ There are also clear opportunities for civil society and vulnerable and marginalised groups to be more involved in HTAs in those countries where HTA does inform HBP decision making, as identified in the WHO report on the broader findings of this HTA/HBP survey.^30^

It is also important to acknowledge that the inclusion of abortion within health financing mechanisms will not be sufficient to ensure access to high-quality abortion care. Beyond issues of affordability, barriers to high-quality abortion care include unnecessary legal and policy restrictions, low availability of services, limited facility readiness to provide high-quality abortion care and abortion-related stigma.^31^ As highlighted in previous research, inclusion of abortion within HBPs does not translate immediately to adequate service coverage. Even where abortion is legal on a broad range of grounds and included within universal health coverage, barriers such as low knowledge, ambiguous laws, conscientious objection, provider bias and requests for unofficial fees can limit access to patient-centred abortion care.^32 33^ In this study, even where abortion was included within the HBP, there were additional restrictions on abortion in some countries, such as inclusion being dependent on the patient’s reason for seeking care. To ensure access to affordable abortion care, these types of restrictions must be avoided, and future research should further assess the conditions attached to inclusion of abortion within health financing mechanisms. With growing use of medication abortion outside of facility settings,^34 35^ it will also be critical for health financing schemes to include this model of care by adequately including abortion medications.

This study has several limitations. The survey only asked respondents about service-specific inclusion within the largest government health financing scheme, so financial coverage of abortion may be higher if it is included within smaller schemes or in a separate vertical SRH programme, as was the case in Nepal and Chile. HBPs are not always explicit, which may have also introduced some inaccuracy into reporting of service-specific inclusion. Although this study is the most comprehensive assessment of HBP abortion inclusion to date, 42% did not respond, and there was lower response from countries and areas in North Africa, West Asia, the Caribbean and Oceania. Many countries in these regions have more restrictive abortion laws,^3^ so non-response may have positively biased the proportion of countries that include abortion in their HBP. Survey respondents were nominated experts from relevant ministries, and they were encouraged to seek input from other colleagues if needed. However, there may have been inaccuracy introduced by survey respondents lacking familiarity with SRH. Responses were not individually validated unless they appeared as extreme outliers, but respondents were given an opportunity to review and update a copy of their response if needed. The survey did not explicitly ask which population groups are covered for each service, and in some cases, the largest government financing scheme only covered a small proportion of the population. The survey did not ask which legal indications are included in the case of abortion, though this information was included in the free-text responses of some countries. The survey did not account for variation in HBP inclusion of abortion by jurisdiction, which is relevant for some countries (eg, UK of Great Britain and Northern Ireland, USA and Mexico). There were high levels of missing data for the assessment of HBP inclusion of abortion by HBP design process variables, which will have introduced unobservable bias, and most of these HBP design process variables were not asked with SRH services in mind (none were asked with abortion specifically in mind). The survey tool did not provide an explicit definition of SRH services, and maternal health was not included within the SRH section, so the question about SRH service exclusion may have been understood differently in different contexts. Finally, categorisation of countries by legal status of abortion is always challenging,^36^ and there are some anomalous cases within the categorisation. For example, a country where the only legal grounds for abortion are life and rape has been included in the ‘grounds specified I’ category, but in practice, this country might be more restrictive than a country in ‘grounds specified II’, which has health as a legal ground for abortion. The law may also be less restrictive in practice than depicted in this analysis. This may explain why, for example, Senegal and Sierra Leone reported abortion is included within their HBP despite having no legal grounds for abortion, as in fact both countries include provisions for abortion within their codes of medical ethics.^37^

However, the study also has several strengths. It provides a comprehensive and timely assessment of the inclusion of abortion within HBPs, including more countries than any previous assessment (80% of the global population), and covering a range of regions and legal contexts. The survey also included HBP inclusion of abortion medications, which has not been assessed in previous studies. The survey included a wide range of SRH programme areas, which enabled systematic comparison between HBP inclusion of abortion and of other SRH services, and an assessment of reasons for exclusion of SRH services. The detailed nature of the survey also allowed for a more nuanced assessment of HBP inclusion of abortion, considering potential conditions of inclusion within the scheme. Finally, survey questions about the HBP design process within the survey facilitated some assessment of how HBP design may influence HBP inclusion of abortion.

## Conclusions

This study provides the most thorough overview of financial coverage for abortion-related care to date, based on responses from 114 countries. The findings indicate that abortion is often not included within HBPs, while treatment for complications of unsafe abortion is more commonly included. Given the substantially higher health system and patient costs associated with postabortion care compared with induced abortion care, this highlights an opportunity to reduce health system expenditure and reduce the impact of unsafe abortion. Political considerations, legal restrictions and stigma may be influencing decisions to exclude abortion from HBPs. HTAs and evidence-based priority-setting, inclusive engagement of stakeholders and transparent priority-setting criteria may support future integration of SRH services within HBPs.

## Data Availability

Data are available upon reasonable request. The data that support the findings of this study are available from the corresponding author, BG, upon reasonable request.
