# Supplementary material for: Inclusion of abortion-related care in national health benefit packages: results from a WHO global survey
Source: BMJ Glob Health. 2023 Aug 29;8(Suppl 4):e012321. doi: 10.1136/bmjgh-2023-012321 (PMC10465905; doi:10.1136/bmjgh-2023-012321)
Supplement: Supplementary data [file bmjgh-2023-012321supp001.pdf]

Appendix 1.

Appendix 1. Countries and areas report of whether any induced abortion is included in their largest public-financed scheme, by the legal status of abortion

	Include any induced abortion	Do not include any induced abortion
**On request**	Argentina, Belarus, Belgium, Cabo Verde, Croatia, Cuba, Czechia, Germany, Guyana, Iceland, Latvia, Mozambique, North Macedonia, Portugal, Serbia, Slovakia, South Africa, Spain, Sweden, Tunisia, Uruguay	Bulgaria, China, Kyrgyzstan, Mongolia, Nepal, Norway, Republic of Moldova, San Marino, Singapore, Thailand, Ukraine
**Grounds specified I**	Bhutan, Chad, Chile, Ethiopia, Gabon, Ghana, Indonesia, Iran (Islamic Republic of), Kuwait, Lao People's Democratic Republic, Liberia, Maldives, Mauritius, Oman, Poland, Republic of Korea, Romania, Rwanda, Timor-Leste, Trinidad and Tobago, United Kingdom of Great Britain and Northern Ireland	Austria, Belize, Benin, Bolivia (Plurinational State of), Brazil, Burkina Faso, Colombia, Côte d'Ivoire, Cyprus, Democratic Republic of the Congo, Ecuador, Eritrea, Eswatini, Gambia, India, Italy, Malaysia, Mali, Namibia, Netherlands, Panama, Tajikistan, United Arab Emirates, Zambia
**Grounds specified II**	Comoros, Costa Rica, Peru, Sri Lanka, Switzerland	Algeria, Bangladesh, Burundi, Lebanon, Malawi, Mauritania, Morocco, Myanmar, Nigeria, occupied Palestinian territory, including east Jerusalem, Papua New Guinea, Paraguay, Solomon Islands, Somalia, Syrian Arab Republic, Tuvalu, United Republic of Tanzania
**No grounds specified**	Senegal, Sierra Leone	Dominican Republic, El Salvador, Honduras, Jamaica, Malta, Nicaragua, Philippines
**Varies by jurisdiction**	Mexico	Micronesia (Federated States of), United States of America

Appendix 2. HBP inclusion of SRH medications in the largest government-financed scheme (n=114)

	Oxytocin		Magnesium sulphate		Folic acid		Misoprostol		Mifepristone		Combi-pack	
	n	%	n	%	n	%	n	%	n	%	n	%
**Fully included**	75	66	71	62	72	63	48	42	25	22	25	22
**Partially included**	15	13	14	12	21	18	16	14	7	6	7	6
**Not included**	6	5	8	7	7	6	16	14	27	24	25	22
**Missing**	18	16	21	18	14	12	34	30	55	48	57	50

Appendix 3. Free-text responses to questions about HBP inclusion of abortion, for 27 countries and areas that provided additional information

Abortion is covered by a SRH programme or national family planning service	Chile	These interventions are not specifically covered by any GES [health insurance]. They are implicitly covered by the public sector through the sexual and reproductive health program. https://www.minsal.cl/portal/url/item/b53faf5d1d527a52e04001011e010ef5.pdf; In Chile, the interruption of pregnancy is allowed under three grounds: the unfeasibility of the fetus, danger of the mother's life or rape. For the first two, there is no limit on the gestation weeks of pregnancy.
	Nepal	All these listed services are part of National Family Planning services which is free. Only services listed under benefit package of social health insurance have been provided.
Additional conditions for abortion inclusion noted	Maldives	Abortions covered for those medically indicated terminations
	Latvia	Induced abortion (medical or surgical) is paid for up to 12 weeks if the pregnancy has occurred as a result of rape.<br>Due to medical indications, a state-paid abortion is performed for up to 24 weeks of pregnancy.
	North Macedonia	Only for abortion with medical indications
	occupied Palestinian territory, including east Jerusalem	Induced abortion (medical or surgical) up to 12 weeks: Applicable. Intervention: - It is done in the case of a justified medical reason.<br>Induced abortion (medical or surgical) after 12 weeks: Applicable. Intervention: - It is done in the case of a justified medical reason.<br>Incomplete Abortion Care: Applicable. Intervention: Depending on the case of abortion, the woman is treated by emptying the uterus, either medically with medications or surgically with a cleaning process to avoid bleeding that threatens life. The woman is subject to monitoring and follow-up either inside or outside the hospital, according to the case.<br>Care for complications of unsafe abortion: Applicable. Intervention: hospitalization and treatment (either a treatment with certain medications or a cleaning process to avoid a life-threatening hemorrhage).
	Peru	Abortions are covered up to 22 weeks only for therapeutic reasons when it is the only way to save the life of the pregnant woman according to article 119 of the penal code.
	Poland	Abortion is covered (regardless of time):<br>(1) when the woman's life or health is endangered by the continuation of pregnancy,<br>(2) when the pregnancy is a result of a criminal act,<br>(3) when there is a high probability of a severe and irreversible fetal impairment.<br>Since the constitutional court ruled that abortion due to fetal defects is unconstitutional the third criterion will no longer be effective after the publication of that sentence.
	Slovakia	Health insurance funds covers abortions in case of health reasons, only.
	Tunisia	In public health structures (ministry of health)
	Ukraine	Abortions only for medical and social reasons
Link to an information page about abortion or legal status clarified	Brazil	Legal abortion is only allowed in some cases as a risk of life to the mother or rape; https://agenciabrasil.ebc.com.br/saude/noticia/2020-09/saude-atualiza-novamente-procedimentos-para-aborto- no-sus; https://elpais.com/sociedad/2020-09-01/los-abortos-legales-en-brasil-se-disparan-durante-la-pandemia-y-exponen-el-drama-de-la -sexual-violence.html
	Colombia	https://www.minsalud.gov.co/Paginas/Precisiones-en-cuanto-a-postura-del-Minsalud-sobre-la-IVE.aspx
	Costa Rica	https://semanariouniversidad.com/ultima-hora/ccss-avala-aborto-farmacologico-en-protocolo-para-interrupciones-de-embarazos-de-riesgo/
	Cuba	https://latfem.org/aborto-en-cuba-mas-de-medio-siglo-legal-y-sin-tabues/
	Guyana	Abortion is legal in Guyana since 1995; https://www.mujeresenred.net/spip.php?article186
	Honduras	https://safe2choose.org/es/abortion-information/countries/honduras#:~:text=El%20aborto%20en%20Honduras%20es%20ilegal%20en%20todas,el%20feto%20no%20puede%20sobrevivir%20fuera%20del%20%C3%BAtero.
	Jamaica	https://www.jalands.org/media/opinion-pieces/2021/2/16/reproductive-rights-in-jamaica-abortion
	Mexico	https://clinicas-aborto.com.mx/me-pueden-atender-en-cualquier-clinica-del-imss-para-abortar/
	Panama	Permitted abortions are for legal reasons or medical indication; https://www.saludmesoamerica.org/sites/default/files/2018-05/4.%20Programa%20Nacional%20de%20Salud%20Sexual%20y%20Reproductiva%20-% 20Normas% 20T% C3% A9cnicas% 20Panama.pdf

	Paraguay	https://www.mspbs.gov.py/portal/15132/solo-se-permite-el-aborto-para-salvar-la-vida-de-una-mujer-afirma-morinigo.html
	San Marino	abortion is not foreseen by our law
No documented information found	Bolivia (Plurinational State of)	No documented information on explicit coverage of interventions 1 and 2 was found
	El Salvador	Abortion is prohibited in El Salvador. No documented information on the coverage of interventions 3 and 4 was found; https://www.telam.com.ar/notas/202105/555128-nayib-bukele-criminalizacion-aborto-el-salvador.html#:~:text= The% 20Salvador% 20es% 20one% 20of% 20the% 20five% 20States, to% 20a% 20ni% C3% B1a% 20a% 20 undergo% 20a% 20a% 20 abortion. https://safe2choose.org/es/abortion-information/countries/el-salvador/
	Dominican Republic	No information was found on explicit coverage of the interventions
Other	Lao People's Democratic Republic	The package covers only treatment, medicine, medical device and consultation
	Malawi	Not Covered
